# Zika: what we know and don't know

**DOI:** 10.11604/pamj.2016.24.33.9749

**Published:** 2016-05-09

**Authors:** Doudou Diop, Dirga Sakti Rambe, Melvin Sanicas

**Affiliations:** 1 EPLS Biomedical Research Center, Saint-Louis, Senegal; 2 Faculty of Medicine of University of Indonesia, Jakarta, Indonesia; 3 Bill and Melinda Gates Foundation, Seattle, WA, USA

**Keywords:** Zika, virus, disease, prevention, treatment

## Abstract

Since the initial reports of a link between Zika and microcephaly, researchers across the world began working toward understanding the virus. In a short amount of time, Zika has become a household name, prompting worldwide concern. The virus is spread rapidly by mosquito bites. We currently do not have a vaccine for Zika. But with the recent findings, vaccine companies are mobilizing their resources to expedite efforts to shave years off the typical decade-long process of vaccine development.

## Brief

The World Health Organization (WHO) has confirmed recently in a scientific consensus that the Zika virus is linked with microcephaly as well as Guillain-Barré syndrome [1]. Previously, the organization had said that there was not yet enough scientific evidence to conclude that the virus caused these conditions, although it was likely. The virus has infected hundreds of thousands of people in 62 countries and territories worldwide and 6 countries (Argentina, Chile, France, Italy, New Zealand and the U.S.) are reporting locally acquired infections through sexual transmission [1]. Nearly 400 cases of Guillain-Barré syndrome in patients with confirmed or suspected Zika virus infection were reported in 13 countries [1]. A new disorder that attacks the brain and spinal cord was recently associated with Zika in adults: an autoimmune syndrome called acute disseminated encephalomyelitis (ADEM) [2]. This finding suggests that the virus may have different effects on the brain and adds to the growing list of neurological damage associated with the virus. A global map calculating when and where the virus is likely to spread shows over 2 billion people could be in the Zika zone [3]. According to the U.S. CDC, the virus is “scarier than we initially thought” and could be linked to more birth defects than previously believed [4]. Meanwhile, the Federal Government lobbied Congress for $1.9 billion to combat Zika [4]. How might that help? How long will it take to develop a Zika vaccine and where might it come from? A report published in Science announced that an image of the 3D structure of the Zika virus has been revealed [5]. Its spherical structure is said to resemble other viruses like the dengue virus; but it also contains differences, such as the dissimilarity of its outer shell [5]. The protein difference found on the virus’ outer shell could explain why Zika attacks nerve cells and that information could lead to discovery of better tools to diagnose the disease or a vaccine. The virus appears to have less diversity among its strains than we see for other viruses. This and the knowledge gained from dengue vaccine development could help expedite the development of a Zika vaccine. WHO launched a global Strategic Response Framework and Joint Operations Plan to fight the virus and the neonatal damage associated with it [6]. WHO says $56 million is required to implement the plan, of which $25 million would fund its Regional Office for the Americas response and $31 million would fund the work of key partners [6]. This figure does not include the total cost for vaccine development - estimated to be between U.S. $200 million and $500 million per vaccine [7]. Financial resource is not the only hurdle in vaccine development - other issues such as ethics, regulatory affairs, protocol design may impede the process especially for this virus that is clearly affecting pregnant women.

Until recently, Zika was labeled “non-threatening”, “rare” and “relatively benign” and there was little incentive for companies to fund research and development of a vaccine. In addition, vaccine manufacturers are usually not keen to spend millions of dollars needed to bring a vaccine candidate from animal testing to human trials unless a market is guaranteed. Now, researchers all over the world have started the process. Currently, 60 companies and research institutions are working on Zika - this includes 18 vaccine candidates targeted to women of childbearing age [8]. Sanofi Pasteur for instance is mobilizing its experts and scientific collaborators to expedite efforts toward the development of a vaccine [9]. The Butantan Institute in Brazil has developed an experimental “inactivated” vaccine, based on killed, purified Zika virus [10]. Bharat Biotech in India announced that it has two candidate vaccines ready for pre-clinical trials [11]. According to the US National Institute of Allergy and Infectious Diseases, a DNA-based vaccine is ready for safety and immunogenicity study [12]. Companies that may have solutions to emerging illnesses experience a rush in the stock market. In 2014, Ebola pushed up the stocks of many companies working on treatments. The same things is happening with Zika - Intrexon, Cerus and Inovio Pharmaceuticals are all working on methods for preventing or treating the virus and all have experienced a boost in their stock prices [13]. However, it is important to keep things in perspective. Epidemic threats do not always turn into profits for developers. Take, for example, the Ebola outbreak, which was controlled before a vaccine was developed. Vaccine manufacturers are also dependent on global governments to buy their product and if we look at Ebola, the countries affected do not have the resources to purchase the vaccines. Aside from vaccines, there are alternative options in fighting Zika. A team of UK researchers has developed a strain of genetically modified male mosquitoes that can mate with females, but only produce offspring that will soon die out [14]. The male mosquitoes have been genetically modified to require a dietary supplement that cannot be found in nature, and the gene is always passed on to their offspring. That means less mosquitoes and less risk of transmission! Given the current status of knowledge of Zika virus infection, clinical progression and pathogenesis, it is not certain where there is a role for therapeutic products. As our understanding of the disease progresses through research, we may discover a role in specific target groups, potentially including congenitally infected infants, long-term carriers and people with autoimmune diseases. Moreover, assuming a vaccine will be available soon, it is currently not clear who should get the vaccine. Should it be given to pregnant women? Should be given to all women of child-bearing age? Because men infected with Zika can have the virus in their semen or spread Zika through sex, should they be vaccinated as well? These are some of the questions that remain unanswered.

## References

[1] World Health Organization (2016). Zika virus microcephaly and Guillain-Barré Syndrome. Situation report.

[2] National Multiple Sclerosis Society Zika virus linked to acute disseminated encephalomyelitis, a disorder that can be confused with MS.

[3] Messina JP, Kraemer MUG, Brady OJ, Pigott DM (2016). Mapping global environmental suitability for Zika virus. Elife..

[4] Center for Diseases Control and Prevention “Scarier than we initially thought”: CDC sounds warning on Zika virus.

[5] Sirohi D, Chen Z, Sun L, Klose T (2016). The 3.8 Å resolution cryo-EM structure of Zika virus. Science..

[6] World Health Organization Zika Outbreak: WHO's Global Emergency Response Plan.

[7] Andre FE (2002). How the research-based industry approaches vaccine development and establishes priorities. Dev Biol (Basel).

[8] Flavia Bustreo Vaccines: a global health success story that keeps us on our toes.

[9] Sanofi-Pasteur Sanofi-Pasteur to leverage its strong vaccine legacy in hunt for Zika vaccine.

[10] Cruz EP Zika: Butantan estuda vacina e soro antivirus, diz secretàrio de Saùde de SP.

[11] Siddiqui Z Barhat Biotech says working on two possible Zika vaccines.

[12] National Institutes of Health Zika virus: a pandemic in progress.

[13] Weintraub A Zika on Wall Street: can these companies really cash in on the virus?.

[14] Creese C Expansion of Oxitec's vector control solution in Brazil attacking source of Zika virus and Dengue fever after positive program results.

